# Cultural Dissonance: Surfers' Environmental Attitudes and Actions

**DOI:** 10.3389/fspor.2021.695048

**Published:** 2021-09-16

**Authors:** Tommy Langseth, Adam Vyff

**Affiliations:** Department of Sports, Physical Education and Outdoor Studies, Faculty of Humanities, Sports and Educational Sciences, University College of Southeast Norway, Bø, Norway

**Keywords:** surfing, sustainability, cognitive dissonance, cultural dissonance, attitude-action gap

## Abstract

Surfers often see themselves as “green”. In this study we examine Norwegian surfers' attitudes and actions towards the environment. The article is based on a questionnaire (*n* = 251) and six qualitative interviews. The results show that most surfers see themselves as environmentally conscious. Oppositely, the data also show that they also buy a lot of surf-related apparel and equipment and travel a lot, and thereby contribute with a lot of CO_2_-emissions. In the article we investigate the apparent attitude-action gap amongst surfers. Does the gap give rise to emotional conflicts? And, if so, to what degree and how do they cope with it. In the article we start out by analysing such potential conflicts by using the concept cognitive dissonance. Further, we analyse the phenomena from a cultural, Bourdieusian perspective where values within the surf-field is highlighted. On the one hand, surf culture highly values connexion to nature and “green” thinking, on the other hand it also values and gives recognition to surfers that travels to and explore exotic destinations. Hence, values within surf culture leads surfers to conflicting actions. We end the article by discussing if these conflicts could be framed as cultural dissonance.

## Introduction

In surf magazines, surf movies, social media and surf literature, surfing is often portrayed as an activity that creates a profound, unique relation with nature—a relation so deep that it almost per se makes surfers environmental stewards (Kampion, 2003; Warshaw, 2003; Ford and Brown, 2006; Hill and Abbott, 2009a; Laderman, 2015). Surfers are depicted as environmentally conscious, and surfing is perceived as a nature-friendly lifestyle. The surfscape is often thought of as an escape from culture into nature (Fiske, 1989; Kampion, 2003). The most influential surfer of all time, Kelly Slater, says that protecting the natural environment is central to surfers' identity (Kampion, 2003, p. 165). Seemingly, being “green” is deeply entrenched in surf culture. At the same time, surfers' quest for perfect waves in tropical paradises and consumption of surf goods, such as surfboards, and wetsuits are also firmly rooted in the surf culture. Wheaton (2020, p. 173) states that “… it seems apparent that debates about surfers as model environmental citizens need reframing”. Latourrette (2005) argues that most surfers remain uncritical of travel and the consumption of surf related products. There is a seeming discrepancy between the surfers self-identification as environmental wardens and their actual environmental behaviour. In a study on surfers in Florida, Hill and Abbott (2009b, p. 157) find that

(…) *surfers generally self-identify as ecologically aware and socially active for environmental protection, reflecting the popular representations of the surfing community. In contrast, analysis of respondents' activities reveals lifestyles often incongruent with environmentally progressive ideologies and practise*.

Surfers' lifestyle, understood through their lust for travel and consumption of surf products, seems to be at odds with their self-acclaimed “ecological” lifestyle. As commonly known, travel, especially a long-haul flight, comes with a huge carbon footprint. The manufacturing of surfboards and wetsuits also involves using a lot of non-biodegradable chemicals (Gibson and Warren, 2014). As Hill and Abbott (2009a, p. 287) state, when the required resources are considered, it is difficult to maintain that surfing has little ecological impact. The question then is how surfers come to terms with the discrepancies between the narratives of surfers as environmentally responsible and their own behaviour? In this article, we explore such tensions in Norwegian surf culture(s). We set out by asking if surfers in Norway consider themselves environmentally conscious—to what degree do they demonstrate environmentally (in the meaning eco friendly) inclined attitudes? Second, we investigate the extent to which surfers' environmental attitudes are translated into environmental actions. The results show a gap between surfers' attitudes and actions. Hence, our third and most important research question is to examine how surfers relate to the attitude–action gap. We discuss this research question through two lenses. First, we explore the gap from a social-psychological standpoint that highlights individual feelings towards the mismatch between attitudes and actions. To analyse this mismatch and the surfers' ambivalence towards it, we build on the concept of cognitive dissonance (Festinger, 1957) and the research by environmental psychologist Stoknes (2015). Second, we analyse the attitude–action gap by using sociologist Pierre Bourdieu's understanding of recognition to understand how different sets of values within surf cultures inform both attitudes that are “green” and actions that are not environmentally sustainable. Building on Bourdieu's framework, we use the concept of *cultural dissonance* to make sense of the attitude–action gap.

### Background

Surfing as a pastime has a history that spans centuries, starting in the Polynesian islands. The Polynesian roots of surfing are often romanticised in surf mythology and in surfers' writings about their own histories (e.g., Young, 1994; Kampion and Brown, 2003). However, the most important influence on the surf culture as it is known today, often overlooked in surf mythology, comprises two elements: surfing was connected to the tourist industry and travel at the end of the nineteenth century and the beginning of the twentieth century to popularise Hawaii as a travel destination, and surfing became part of the rising youth culture in the 1950s and the counterculture of the 1960s (Warshaw, 2010). Of course, other significant discursive elements substantially influence present-day surf cultures, but these two components are important in understanding the environmental attitude–action gap. On one hand, surfing is deeply connected to emission-heavy travel and exploration; on the other hand, it holds the environmental attitudes from the 1960s' counterculture.

In Norway, surfing started to gain a foothold in the early 1980s, but it was not until the mid-1990s that a proper surf culture with a certain number of surfers developed (Langseth, 2012). Currently, certain surf spots are regularly surfed all along the Norwegian coast, many of them heavily crowded every time there is a decent swell. As the number of surfers in Norway has increased, so has the attention to issues concerning surfing and the environment. Founded by surfers in 2013, the Nordic Ocean Watch (NOW) can be regarded as the Norwegian equivalent to activist surf organisations, such as the international Surfrider Foundation and the British Surfers Against Sewage. In a study of Surfers Against Sewage, Wheaton (2007, 2008) found that surfers should not merely be viewed as individualistic hedonists, but that such organisations can be seen as New Social Movements that are often apathetic to traditional politics but still environmentally aware. In these movements, political protests often take creative forms. Further, Wheaton (2008) states that surfers often claim to have a privileged relationship with the water. This privileged relationship is often thought of as an impetus to environmental awareness. On its website, NOW states, “We believe that you take care of what you love” (https://nordicoceanwatch.no/en/who-are-we/). However, in an interview published in *Infinitum* magazine (October 2014), NOW founder Simen Knudsen reveals a bit more ambivalence:

*If we love to play in the ocean, and we know that CO*_2_ *is the biggest threat to the ocean, and facts show that we all have to get down to two tonnes of emissions per person per year. How can we then defend flying a flight to the other side of the globe, where the main purpose is surf? Is this in reality institutionalised egoism and what sentiment is it really that we are chasing, that makes us fly across the globe to experience it?*

Seemingly, it is not as easy as claiming that if you love nature and the ocean, you protect them, but what do the research findings reveal about this?

### Surfing and the Environment

According to McCullough et al. (2018), four main discourses have dominated the research on how nature-based sports influence the environment. (1) a discourse around the impacts of nature sports on biophysical properties of ecosystems, (2) discussions on how, among others, the expansion of skiing areas has affected land use, (3) the impacts of tourism on local social systems, and (4) since the 1990s, a discourse on the global impacts of sport travel and the production of sport equipment. These discourses also signal a move from a focus on nature-conservation to a focus on climate-protection. When we discuss the environment and being environmentally aware in this article, we use these concepts as umbrella concepts that involve nature conservation, climate protection, and pollution. That is—we do not directly deal with the environment as materiality in this article. Rather, we focus on how the environment is perceived, given value an acted upon.

Another, parallel discourse has been about whether, and to what extent, participating in nature sports and outdoor activities makes people more concerned about environmental issues than people not participating in such activities. According to the general belief, as people have personal experiences in and with nature, they also take care of it. This claim is also found in research. Olive (2016, p. 503) states:

*With a sense of individual connectivity and experience so key to the development of ecological sensibilities, nature-based sport and physical cultures offer productive and potential space in terms of environmental sustainability*.

However, as Høyem (2020) shows, it is difficult to maintain a causal relation between being in nature and aspirations to protect the environment. In Norway, where outdoor recreation is highly popular and deeply connected to the national identity, these kinds of thoughts have been taken for granted. From the early 1970s and onwards, outdoor recreation in Norway, to some extent, has been connected to the deep ecology movement. Naess (1973), a Norwegian philosopher who coined the term *deep ecology*, was an avid rock climber and outdoor enthusiast. Although most Norwegians participating in outdoor recreation most probably have not read Næss' work, he has deeply influenced the Norwegian discourse around outdoor recreation (Gurholt, 2008). This might have influenced the thought that just being in nature (in the correct ways) leads to behaviour that is good for nature. However, as shown by Hille et al. (2007) in their study on leisure consumption, traditional outdoor sports, such as cross-country skiing and hiking, had the third highest energy use out of the leisure activities they examined. Outdoor activities were behind holiday travels and visits to friends and relatives but had three times the energy consumption of traditional sports (Hille et al., 2007, p. 164). The reasons for the high energy consumption connected to outdoor recreation are the amount of travel to outdoor areas and the use of outdoor-specific clothing and gear. Seemingly, at least from the perspective of the above-mentioned fourth discourse on sports and the environment, there is not much to the argument that spending time in nature leads to environmentally sound behaviour. Being an active outdoor recreationist is actually among the worst leisure activities people can participate in when it comes to energy use and CO_2_-emissions. Concerning the energy use in outdoor recreation, Aall et al. (2011, p. 467) state, “We have also found that the large increase in negative environmental impacts of leisure activities can be characterised by the proverb ‘the road to hell is paved with good intentions'.” They find that experiencing nature (seeing new places, etc.) has “unfortunate side effects in also acting as drivers for producing more negative environmental impacts when applied to leisure activities (*ibid*, p. 467). In other words, experiencing nature does not necessarily entail environmental behaviour. However, this is complex matters—studies of place-attachment have come to different conclusions. Some studies have shown that place-attachment leads to more pro-environmental behaviour (Vaske and Kobrin, 2001) while others have found it to be associated with less pro-environmental behaviour (Uzzell et al., 2002). Place-attachment might be a too wide concept to study environmental behaviour. When Junot et al. (2018) narrowed the concept down to place *dependency*, how people perceive that they are dependent on a certain environment for their own well-being, they found that it corresponded to environmental behaviour. Still, even if people state that they are concerned about the environment and that they turn off the light when they leave a room and compost their kitchen waste, that doesn't necessarily translate into an environmentally sound lifestyle. These environmental values are, we suppose, typical middle-class values, and at the same time it is the middle class that has the highest energy use connected to leisure activities. Composting kitchen waste doesn't really help much compared if you take the family on two annual trips to Thailand. Larson et al. (2011, p. 68) claim that, “A growing body of research shows that positive exposure to nature through outdoor recreation participation may contribute to a pro-environmental ethos.” The problem is that having an environmental *ethos* does not, as said, necessarily entail actions that are environmentally sound. In a study of mountain bikers in the US, Barnes (2009) found that some environmentalists were taken by surprise by the deteriorating effect their activities had on the landscape. Further, Wilson and Millington (2013) suggest that some wilderness activities are based on deceptive associations with environmentalism. More specific to surfing, Booth (2020, p. 26) states that “…a large body of evidence challenges the notion of surfers as active environmentalists who are willing to protect coastal environments because of their relational sensibilities with the waves”. Moreover, As shown by Hille et al. (2007) and Aall et al. (2011), partaking in outdoor recreation, even though it might entail an environmental ethos, is actually one of the least favourable pastimes that people can engage in, concerning their ecological impact. The same can probably be said about surfing. Borne (2018) asks if surfers are more environmentally aware than the general population because of their close contact with nature. His own answer is “unfortunately there is little evidence to support this proposition” (Borne, 2018, p. 53). Surfers in general have high environmental engagement, but statistics show their much higher carbon footprint than that of the general population (Butt, 2015). In both surfing and outdoor activities in general, there seems to be a gap between the participants' concern for the environment and pro-environmental behaviour (Stoddart, 2011; Butt, 2015; Evers, 2019; Wicker, 2019).

As Berns and Simpson (2009) note, an array of concepts is used in studies on environmentalism. In this article, we use the concepts of environmental *attitudes* and environmental *actions* to differentiate between people's environmental values, ethos, and concern, on one hand, and actions that have beneficial impacts on the environment, on the other hand. Hill and Abbott draw the boundaries between the two concepts in this manner:

*Opinions expressing the general responsibility of individuals and groups to maintain the quality and function of ecological systems are environmental attitudes. Environmental actions are activities that people engage in with the intent to preserve or enhance ecological systems. People may agree with environmental attitudes without actualizing these beliefs through environmental actions* (2009b, p. 158).

## Materials and Methods

To get a grip of surfers' environmental attitudes, actions and ambivalences, we rely on two sets of data: one quantitative dataset based on an online survey (*n* = 251) and six qualitative semi-structured interviews.

To sample data from a wide range of Norwegian surfers, a survey was chosen as the preferred method. This allowed us to let our data be in dialogue with and complement Hill and Abbott's study of surfers in Florida (2009a; 2009b). For the survey, we used the Questback software. The survey was distributed through different Norwegian surf-related Facebook pages. As Whitaker et al., (2017) points out, recruitment through Facebook is a cost-effective recruitment tool. 251 surfers responded to the survey of which 19% identified as women and 81% as men. The majority of the respondents were between 18 and 39 years old, with 58, 28, 11, and 2% belonging to the 18–29, 30–39, 40–59 and 10–17 age ranges, respectively. The low number of surfers under 18 years old might seem surprising but is probably due to the requirement to have a driver's licence to be a surfer in Norway. As a young surf culture, not many young people have parents who are also surfers and willing to drive them to the beach to surf. Geographically, most parts of Norway were represented, from Kristiansand in the south to Lofoten in the north. Southwestern Norway was over-represented, but this is also probably the area with the highest density of surfers in the country (Langseth, 2012). However, self-recruitment *via* Facebook might have its downsides. One consequence of this method is that surfers who do not use Facebook are not represented in the survey. Also, as Lazarow and Olive (2017) notes, self-selection can lead to a bias towards core participants. In our case this could also mean that our respondents are surfers that are more concerned about the environment than the average surfer. However, in a study of Facebook surveys compared to traditional surveys, Kalimeri et al. (2020) found that biases was negligible. How and if this recruitment method has influenced our results is hard to know. Nonetheless, we think that the effectiveness and advantages of using such an approach outweigh potential biases.

The questionnaire items were divided between attitude questions and action questions. The questionnaire was set up in such a way that when the respondents had answered one set of questions, they could not go back to change their answers. Our reason was to prevent the respondents from changing their answers to the attitude questions if the action questions revealed to them their lack of environment-friendly behaviour even if they had stated their environmental inclination. The question whether the surfers considered themselves to be environmentally conscious was deliberately loosely defined. From our side we didn't want to state if we thought about nature conservation, pollution or climate protection. We used a vaguely defined term to reveal what the informants themselves think when such a term is used.

To gain a deeper understanding of the attitude–action gap, especially the potential ambivalences resulting from this gap, we relied on semi-structured interviews (Kvale and Brinkmann, 2009) conducted among surfers living on the Lofoten Islands in northern Norway. Lofoten Islands is one of the most popular surf destinations in Norway and probably the Norwegian surf area that has received the most attention in the international surf media. Hence, many travelling surfers surf in Lofoten. However, the informants who were recruited for this project were either permanent or seasonal residents of Lofoten and therefore well integrated in the local surf culture. The interviews followed a guide containing questions and issues concerning surfing and environmental matters. The interview guide was not strictly followed, allowing a departure from it if interesting themes merged that we had not thought of when developing the guide. Four men and two women were interviewed. Their ages ranged between 21 and 41 years. The interviews lasted between 70 and 140 min each, which were all recorded and fully transcribed.

As we are both surfers, we might run the risk of just accepting other surfers' storeys of themselves as environmental stewards. To avoid this, we adopted an analytic strategy that we could call a “theoretically informed reading of the results.” The theoretical framework used in this article has been applied to both the construction of the questionnaire and the interview guide and the analysis of our data. This means that we use theoretical concepts as tools to create distance and to objectify the empirical data. It also indicates that we reject ideas of a “holistic” approach to the gathered materials and refuse taking interviewees' statements at face value. Rather, our aim is to remain critical of and distanced from our data in order to gain insights into the theme of surfing and sustainability.

We gathered the data in accordance with the guidelines of the Norwegian National Committee for Research Ethics in the Social Sciences and the Humanities (NESH). The answers to the questionnaire were not traceable to any names or IP addresses. All interviewees received written information about informed consent before agreeing to be interviewed. The transcribed material was anonymised and safely stored in a secure database after the interview process.

## Theoretical Approach

In this article, we approach the attitude–action gap from two different angles. One angle lets us highlight the individual ambivalences that surfers might experience in relation to their environmental attitudes and actions; the other helps us develop an understanding of how the value system in surfing gives surfers recognition for being “green” on the one hand and extended travelling on the other. These values thereby give surfers impetus for both pro-environmental attitudes *and* actions that might not be pro-environmental. From the former perspective, we use social psychologist Leon Festinger's theory of cognitive dissonance (1957) and environmental psychology. From the latter perspective, we apply Bourdieu (1977, 1990) conceptual framework to get a grip of values, and recognition in surf cultures.

Festinger (1957) is well known for his concept of cognitive dissonance. Broadly speaking, Festinger's theory is an attempt to describe how contradictions between convictions and behaviour cause unpleasant mental tensions. More specifically, in Festinger's framework, the concept of cognitive dissonance is coupled with cognitive consonance. Festinger divides human attitudes and actions into three parts: affective, cognitive, and behavioural components. If there is concordance among these components, Festinger calls it cognitive consonance. He holds that this is the state that we strive for as human beings (1957, p. 3). A discrepancy between these components will lead to cognitive dissonance. People will then try to alter these components so that they return to a state of consonance. In other words, cognitive dissonance describes an unpleasant and unstable mental condition that is experienced when an individual's thoughts and values do not match one's actions. In environmental psychology, cognitive dissonance has been used to describe what occurs when environmentally conscious people find out that their behaviour is not environmentally sustainable. They can either change their behaviour or create consonance by legitimising their behaviour in one way or another (Lavergne and Pelletier, 2015; Stoknes, 2015).

A question to Festinger could be: do people necessarily experience unpleasant emotions when there is a discrepancy between their attitudes and actions? Moreover, is the attitude–action gap just a cognitive dilemma? A foundational thought in sociology is that individual actions and thoughts are connected to the social groups to which the individual belongs. To grasp how surf culture influences attitudes and actions, we now turn to Bourdieu (1977, 1990, 1999) understanding of social practise and processes of recognition.

As Bourdieu's perspective is well known and often used in the sociology of lifestyle sports (see Laberge and Kay, 2002; Wheaton and Beal, 2003; Fletcher, 2008; Atencio et al., 2009; Thorpe, 2009; Langseth, 2012; Langseth and Salvesen, 2018; Tøstesen and Langseth, 2021), we do not elaborate Bourdieu's framework in detail here. Building on Langseth (2012), we use Bourdieu to understand how surfers, through subcultural socialisation processes and accumulation of surf-specific forms of capital, develop strong affectual relations with central values in the surf culture. Nonetheless, a brief explanation of Bourdieu's lesser-known understanding of recognition and the link between recognition and desire is needed.

Recognition is a key term in sociology and social philosophy. Axel Honneth's “Struggle for recognition” (2007) and Charles Taylor's “The politics of recognition” (1994) are perhaps the most central works in modern recognition theory. Bourdieu has to a lesser extent been central in recognition discussions. Whereas Honneth and Turner are strongly normative—they are concerned with how mechanisms of recognition *should* work to create the most just society—Bourdieu's understanding of recognition is connected to how recognition actually works in specific social groups. In Pascalian Meditations (1999, p. 173), Bourdieu argues that the pursuit of recognition is the driving force behind all investments actors make in a field. In other words, Bourdieu sees recognition as fundamental to a field's capital system. Recognition is what both lays the foundation for the existence of a field and what actors within the field strive for. Building on Bourdieu, Crossley (2001) argues that desire is connected to recognition. In Crossley's view, what people desire, what they strive for and their passions are connected to what is given recognition in the social environment where they belong. Bourdieu (1996) states that there are just as many forms of libido as there are social fields. All fields have their own norms and rules that should be incorporated into each agent's habitus for the agent to be able to play the game. For a game to function well, the agents have to forget that they are playing a game; the rules of the game have to be viewed as “natural.” According to Bourdieu, incorporating a field's rules is what makes actions appear meaningful (1990, p. 66). The dominating rules of the game are taken for granted; they represent what Bourdieu calls the fields' “doxa” (1990, p. 58). That means that desire and passion is related to being part of a social game. Surfers that fight on the beach over an argue about who stole whose wave, is not the result of antisocial affects, but rather an expression of the surfers' heavily involvement in the surfing game. Even if the fighting is antisocial normatively, it is deeply social because it stems from their investments in the surfing game. Simply put, passion is related to what is given credibility in a certain social environment. The question for us, then, is if attitudes and actions connected to pro-(or con)-environmental behaviour is given recognition within surf cultures. Our research thereby ties with other research that highlights the connexion between emotions, affects and socio-cultural processes, and narratives (see Tamminen and Bennett, 2017) and the connexion between environmental actions and emotions (Carmi et al., 2015).

## Results and Discussion

### Surfers' Environmental Attitudes

As reported by both Hill and Abbott (2009b) and Borne (2018), surfers state that they have environmentally inclined attitudes. Our survey's results, to a large degree, show the same tendency: 84% of the surfers state that they either agree or strongly agree that they perceive themselves as environmentally conscious. Almost nobody disagrees with being environmentally conscious. Another factor that might indicate something about the respondents' care for the environment is that 65% report “experiencing nature” as one of the most important motivations for surfing. Now, this does not necessarily point to environmental attitudes—a person might be interested in nature experiences without being concerned about the environment. Nonetheless, many of the interviewees in the qualitative sample claim that the close relation with nature that surfing provides gives rise to environmental thoughts. “Bjørk” says, “Of course, everyone who resides in and uses nature have a responsibility (…). If you use nature, appreciate it and think it is nice, then you have a responsibility to be good to it.” “Frank” takes it a bit further. For him, spending time in nature does not just give surfers the responsibility to act in environmentally sound ways, but communion with nature is what instils environmental sensibilities in surfers:

*It is clear that as surfers, we are especially close to nature. Compared to football, for instance, the cause is obvious why surfers are more environmentally conscious. It is because we are directly confronted with nature in the activity itself*.

The narrative of how being in the ocean makes surfers environmentally conscious is widespread in our data. This is also found in many other outdoor activities and is discussed in the research literature (e.g., Høyem, 2020). As we mentioned earlier, this narrative is complex and debatable. In the preceding excerpt, Frank states that surfers are more environmentally conscious than football players. That might be the case, but we know that when it comes to environmental actions, at least in terms of energy consumption and CO_2_-emissions, outdoor activities are generally worse for the environment than organised sports (Hille et al., 2007). Let us have a look at how surfers in Norway transfer or try to put their attitudes into action.

### Environmental Actions

As shown in Table 1, the surfers in our survey respond that they are involved in environmental actions to varying degrees. Of the survey participants, 69% report that they have been involved in beach clean-ups, and 50% state that they eat less meat. However, only 18% are members of an environmental organisation. If being a member of an environmental organisation is an expression of attitudes or actions is hard to comprehend, depending on how involved the person is in the organisation. Nonetheless, it might hint that even if surfers consider themselves environmentally conscious, only to a little extent do they get so involved that they choose to become members of such organisations. Perhaps more relevant to this study is the finding that only 23% of the respondents say that they try to reduce their air travel (more about that later). The same pattern is found in the qualitative data. Beach cleans-ups seem to be what surfers are most concerned about. “David” explains:

*I try to think of what is important to do for the environment (…) because you do get a bad conscience sometimes. So, I participate in beach clean-ups with barbeques and things like that—where you pick up waste and it also becomes a social event*.

**Table 1:** Frequency of respondents participating in specific environmentally responsible activities (*n* = 218).

Beach clean ups	69%
Eat less meat	50%
Avoid products containing micro plastic	33%
Avoid red listed seafood	30%
Reduce air travel	23%
Member of an environmental organisation	18%
Other	27%

David clearly regards participating in beach clean-ups as a way of doing something good for the environment. A beach clean-up event does not only seem to be about tidying up the beach but is also more of a personal catharsis as it helps David deal with his feelings of a bad conscience about the environment. Such events also have a social element that seems to be a motivating factor for environmental actions. In contrast, “Clara” explains that she does not need this social dimension or an event to pick up waste from the beach:

*I participate in beach clean-ups sometimes, but it is not there [that] the big effort is made, really. It is rather when you go for a hike or are in the water—when you find waste and take it with you. I do not feel that I have to be at a beach clean-up to pick up waste because I do that anyway*.

The reason behind the popularity of beach clean-ups and picking up garbage is probably that pollution from waste on beaches is concrete and visible. As “Eddy” says, “It is only when you get to the beach and you see waste floating around that you get motivated to do something about it.” Several informants express their stand that it is when their local surf spot becomes affected that they really start doing something for the environment. Compared with other environmental threats, such as carbon dioxide emissions and non-biodegradable chemicals from surfboard and wetsuit production that are abstract and distanced, the waste on the beach is concrete and experienced directly. Evers (2019, p. 424) calls this direct, embodied experience of non-human agents like waste on the beach “polluted leisure”: “The concept of polluted leisure describes the embodied, sensorial, emotional, intellectual, spatial, and technological emergence of pollution—material and social, harmful and nonharmful, actual and perceived assembling with leisure”. In our data, polluted leisure, when experienced directly, when surfers for instance see and feel that the beach is flooded with plastic waste, seems to give direction to environmental actions. Whereas the embodied sensual experience of garbage on the beach might be the reason that so many surfers participate in beach clean ups, other environmental hazards are only known through scientific “black boxing” (Latour, 1987). The technological emergence of pollution does not seem to entail the same actions as those directly experienced. Nevertheless, pollution that are not experienced through the senses, seems to have an impact on the respondents. As seen in Table 1, almost 50% of the respondents say that they eat less meat. This can be perceived as a response to the more abstract threats of climate change. Frank says, “I try to eat less meat. Not necessarily because I am against eating animals [but] more because I try to contribute to limiting the industry and of course, also because of health reasons”. For Frank, apart from thinking about his own health, his reasons for not eating much meat are connected to the meat industry and the industrial production of meat. Similarly, Bjørk says, “I am very concerned with meat production. I don't eat anything that is mass produced. Just whale, reindeer and moose.” Perhaps surprising to international readers, Bjørk views eating whale as part of being environmentally concerned. In most places, this might be regarded as unethical in itself, but she considers it part of eating short-travelled, non-mass-produced meat.

Another question is if these surfers think about environmental issues when they buy surf equipment. Table 2 shows that over half of the surfers in the survey own more than three surfboards. If that is a high number or if it is necessary might be debatable, but it does indicate these surfers' quite high consumption of surfboards. Nearly half of the respondents also own more than three wetsuits. Having two wetsuits can be said to be a necessity in Norway. A person should have a thick wetsuit to surf during the winter months in Norway, but the winter suit is too warm in the summer. Thus, to be a surfer in Norway means that one does need at least two wetsuits. The fact that many surfers have more than that might just be a sign that they use their wetsuits for a long time, even after these are semi worn out. The question then is rather how often these surfers buy new apparel and equipment.

**Table 2:** How many surfboards and wetsuits do you own? (N = 250).

	**Surfboards (%)**	**Wetsuits (%)**
0	6	2
1	20	19
2	22	34
3	15	23
4	14	12
5+	22	9

Over half of the surfers in this survey buy a new surfboard every year or every second year (51%), and almost the same percentage (46%) is found to buy wetsuits within the same time frame (Table 3). Again, the question of whether there is a “need” for these purchases can be discussed. Undoubtedly, the findings indicate a high amount of consumption of products with a substantial content of chemicals and substances that are not exactly eco-friendly. Several manufacturers of surfboards and wetsuits have developed products that are less harmful to the environment. These industry efforts of green consumerism (Erickson, 2011) and ecological modernisation (Wilson and Millington, 2013) does not seem to appeal to the surfers in this survey (Table 4). Nonetheless, when buying new wetsuits, the most important factor, along with specifications (the suit has to fit; otherwise, it will not be warm enough), is durability. What this means is hard to know based on our data. It can mean that the buyers are concerned with economy (they do not want to spend money on wetsuits too often) or with the environment.

**Table 3:** How often do you buy new surfboards and wetsuits? (*N* = 240/243).

	**Surfboards (%)**	**Wetsuits (%)**
More than once a year	5	2
Once a year	22	18
Every second year	24	26
Every third year	19	25
Every fourth year	9	10
Every fifth year +	20	20

**Table 4:** What is most important for you when you buy surfboards and wetsuits? (maximum 2 answers) (*N* = 246/249).

	**Surfboards (%)**	**Wetsuits (%)**
Eco friendly production	12	15
Price	39	33
Brand	7	6
Specifications	74	59
Durability	33	61

Taking flights is probably the surf-related activity that has the largest impact on the environment. As shown in Table 5, most of the surfers in the survey do not fly much domestically, and about one-third do not take any international surf trips annually. However, approximately two-thirds of the respondents admit taking one to two surf trips to Europe and internationally per year, which involve flying. In other words, the average surfer in this survey goes on one or two trips to Europe each year, and one or two trips to destinations farther away. As Borne (2018) points out, emissions from flights are where surfers really stand out as not behaving in an environmentally sound way. David says:

**Table 5:** Flights per year where the purpose of the flight is surfing.

	**0 (%)**	**1–2 (%)**	**3+ (%)**	**N**
Domestic	65	25	10	228
Europe	35	60	5	221
Rest of the world	37	58	6	233

*I fly about six times a year, I think. A couple trips abroad, and a couple domestic. The dilemma is that people who surf also travel a lot via planes. People like to travel to places with good waves, but flying too much is not good for the environment*.

In their study on surfers surfing at spots where surfing is forbidden because of bird nesting, Løland and Langseth (2017) found that surfers continued to surf despite the ban because of the activity's deep importance to them, a part of their identity and habitus. The same phenomenon can be observed here; David explains about extended travelling in the search for high-quality waves. Nevertheless, he also expresses a certain ambivalence towards this—he knows that the quest for high-quality waves is not environmentally sustainable.

### Ambivalence and Dissonance

As we have shown, most surfers consider themselves environmentally conscious. The qualitative interviews provide the same result from all but one informant. At the same time, we observe that their actions, especially regarding surf-related consumption and travel, somewhat oppose their self-image. Hill and Abbott (2009b, p. 160) state that

*even though many surfers consider themselves environmentally progressive, we find that they are incompletely aware of their own environmental impacts, thus limiting their behaviour as environmental stewards*.

Following the same line of thought, Stoddart (2011, p. 20) informs that “These inconsistencies between attitudes and behaviour are not unusual. Rather, they are among the many tensions and paradoxes inherent to social life in the twenty-first century consumer-oriented society.” The question is to which degree surfers are aware of the discrepancy between their attitudes and actions. And—if they are aware of it, how do they deal with this ambivalence? Clara relates to this issue when she says:

*I would say that I am environmentally conscious to the degree that I understand that my actions influence the environment and have an understanding of how bad it can be. I am aware that my actions have negative consequences for the environment. I know that flying somewhere has a cost for the earth because you leave a footprint*.

Many of the informants express similar ambivalences—they know that their actions are not good for the environment. For some, this gives rise to a feeling of a bad conscience. Bjørk states:

*I quickly get a bad conscience when I travel. Maybe not so much by car, more with planes. Because you want so much to travel. But is that really environmentally conscious? And at the same time, the desire to travel is huge***. ***And I think that it is a challenge that many who see themselves as being environmentally conscious have. They also want to travel and are adventurous*.

For Bjørk, surfing and environmental consciousness appear as an utterly ambivalent and complex relation. The desire to travel and experience exotic waves is strong. At the same time, a bad conscience lurks at the back of her mind. This seems common to most of the informants. However, Eddy expresses another view:

*When it comes to surfing, I don't let the environment stand in the way of a flight. Absolutely not. Then I'd take a flight to go surfing. If you have the means to do it—then it is just “off we go”. Then surfing comes ahead of the environment*.

Even if the quotes from Bjørk and Eddy show contrasting feelings of guilt and lack of remorse, they both reveal a passion for surfing that makes them act in ways that are not environmentally sound. For some participants of this study, this seems to lead to what Festinger calls cognitive dissonance. Environmental psychologist Per Stoknes (2015, p. 63) explains, “We have two thoughts, or cognitions: *I have a large carbon footprint*. And I've learned that *CO*_2_ *leads to global warming*. These two notions don't go well together. They conflict with a positive self-image and create a vexing discomfort.” This appears to be exactly what happens to our informants. According to Festinger (1957), to reinstate consonance, people experiencing dissonance have to legitimise their behaviour in some way or another. For instance, Frank says, “Well, I am environmentally conscious, but if I act in an environmentally friendly manner, I don't know. I try to eat less meat. (…) and I always sort my waste.” Eating less meat and participating in beach clean-ups can be perceived as measures that help justify the informants' self-identification as environmentally conscious. To cite another example, David says, “I bought a bike so I can start biking to work. That is being environmentally conscious.” Biking to work and eating less meat can be viewed as simple measures that compensate for the ambivalence and the dissonance that the informants might feel. This can be considered what Wicker (2019) calls “low-cost situations.” Cost, as it is used by Wicker here, is not about economy, but rather what facets of an activity that is seen as more or less important by the participant. Wicker finds that many sport participants engage in low-cost environmental actions, such as separating waste and buying local food. In contrast, it is much harder for athletes to scale down on travelling, which seems to be a high-cost situation—situations that are more important to the athletes. As Lazarow and Olive (2017, p. 215) states “…what surfers are willing to give up to secure better environmental and social conditions is difficult to know, and decisions are often influenced by various social and cultural trends.” However, participating in low-cost situations apparently might be seen as helping surfers get rid of their dissonance. Another way of justifying travelling is by stating that they actually learn a lot about the environment when they travel. David explains:

*You do get to see and experience a lot when you travel. I've been to Indonesia, and I have seen how much plastic floats around. You do get very aware that we as human beings should do something to take care of nature*.

The thought that David expresses here is that even though travelling has environmental impacts, it can be defended because it leads to pro-environmental behaviour in the second instance. It is difficult to determine the degree to which there is any truth to such a claim. Such a statement can nonetheless be interpreted as an expression of David's attempt to justify his actions in order to create cognitive consonance.

To return to the question of high vs. low cost, why should travelling be considered a high cost? As Bjørk says in an above-cited quote, surfers consider themselves ecologically responsible, but they also feel the urge to travel and embark on an adventure. Eddy delves into this self-contradiction when he says:

*Whatever your activities are, these are going to affect the environment in some way or another as long as you are passionate enough. I don't think there are people who give up good experiences because these affect the environment*.

Eddy clearly indicates that for him, and in his opinion, for most people, the passion for surfing outweighs taking care of the environment. As Evers (2019, p. 433) explains, concern about the environment is entangled with other desires and needs. Many of the surfers in our data material are torn between two passions—a passion for the environment and a passion for surfing that entail travelling and consumption of surf-related goods. To further explore this issue, we now turn to the sociology of passion.

### Cultural Dissonance

The theory of cognitive dissonance to a certain degree presupposes that human beings are rational actors; if a person acts in conflicting ways, this must be solved in some way or another. This might be the case for some informants—for instance, they have to defend their travelling by saying or thinking that they do a lot of other good things for the environment. Others may not think of or feel such conflicts. Another question is to which degree these inner conflicts are felt and to which extent they are narratives that interviewees come up with when pressured to talk about the issue in an interview. Anyway—whether these are real feelings or narratives—this research has so far shown a gap between actions and attitudes. The surfers seem to have two conflicting passions; one drives them to perceive themselves as environmentally conscious *and* the other drives them towards actions that are not so good for the environment. To understand the process behind passion development, we have to turn to the value system of surfing, in other words, what is given recognition in the surf culture.

The connexion between value systems, recognition and desire development has previously been described by Langseth and colleagues. They have shown how risk can be perceived as a form of symbolic capital in base-jumping, climbing and freeride skiing, which gives rise to a logic that connects risk-taking, recognition and status (Langseth, 2016; Langseth and Salvesen, 2018; Tøstesen and Langseth, 2021). In a study on Norwegian surfers, Langseth (2012) has found that the dominant forms of symbolic capital are skills, subcultural knowledge, commitment, and local affiliation. According to Langseth, these values determine status and position in the social hierarchy of surfing. The values are gradually learned as neophyte surfers are socialised in the surf culture and become “natural” —taken-for-granted part of surfers' thinking. These values can be regarded as part of the doxa of surfing, values that are naturalised and not questioned (Bourdieu, 1990). The values described by Langseth are of course not exhaustive—the value system of surfing is more extensive than this. The question then is how the attitude–action gap can be understood from this perspective. Let us look at a few quotes. Clara says:

*When I tell people that we have [stayed for] five weeks in Sri Lanka, it is inconceivable for some to travel and be away for such a long time just to surf, but it can impress others. It can be an explanation of why many choose to travel because it is a status symbol to surf in many different places and that you are not narrow-minded and just surf in Unstad*.

Several elements are revealed in this quote. First, when Clara says that many people do not understand why she can go on such extended trips just to surf, we assume that these people are probably non-surfers—persons who do not understand the passion for surfing—and in Bourdieu's view, stand outside the field of surfing and do not comprehend its inherent value system. Second, the quote shows that just surfing on your local spot (in this case, Unstad, Lofoten) is regarded as being narrow-minded. In other words, staying and surfing in your local environment are not what gives recognition. Third, she clearly states that travelling “impresses” people and grants status. She goes on to elaborate on this matter:

*Maybe you have surfed a wave that requires a certain skill level and that everybody knows about. And people just ask, “Have you surfed there?” For your own part, you feel [a sense of] achievement, and I also think that it has to do with status—that you have been around and can impress people in that way*.

Here, Clara expresses two things: surfing famous waves abroad gives a sense of personal achievement *and* grants status and recognition. The Bourdieusian point here is that these two elements are connected; what people want to achieve is linked to what offers them status and recognition in a certain culture. In the value system of the surf culture, travelling is a form of symbolic capital that grants status and recognition. It can be perceived as part of the unquestioned doxa of surfing. Much like in skiing, where participants rely on mobility networks to perform their preferred activity (Stoddart, 2011), surfing is also deeply connected to transport (see Wheaton, 2020). Both in form of car dependency and long-haul flights. The value that travelling holds in the doxa of surfing has a long history, and it would be outside the scope of this article to provide an in-depth analysis of this theme. Ruttenberg and Brosius (2017, p. 111) maintain that “…surf media and industry continue to construct a travel-to-surf narrative….” Aside from media and industry, another element that influence the value of travel in surfing is that modern surfing has always been connected to tourism and travel. After surfing regressed as a pastime in Hawaii in the nineteenth century, its rebirth was to a large degree part of marketing Hawaii as an attractive tourist destination at the end of that century (Warshaw, 2010). Furthermore, when surfing advanced from an activity that had very few participants to become part of the pop culture in the 1950s and the 1960s, movies such as *Endless Summer* (1966) highlighted travel as an essential aspect of what it meant to be a surfer and thereby consolidated travelling as part of the value system of surfing.

The point here is that travelling is so ingrained in the surf culture that even surfers who perceive themselves as “green” cannot and will not avoid it. Instead of understanding the attitude–action gap at just a cognitive level, it is important to comprehend that this discrepancy has deep roots in the cultural history of surfing. Travelling is important to surfers, and so is being “green.” Again, it would require much more space than this article allows to delve into the connexion between surfing and environmentalism. Nonetheless, it is safe to say that being environmentally conscious is also part of the value system of surfing. Stoddart (2011) found that in the discursive construction of skiing, skiing is linked with nature and environmentalism. The same can be said about surfing. Eating less meat, taking part in beach clean-ups and so forth are also actions that give recognition in the surf culture. When a person opens a surf magazine, one will find a lot of information about how important professional surfers think it is to take care of the environment. As mentioned, exploring how environmentalism has come to be part of the value system of surfing would require systematic research on this subject. Nonetheless, the connexion between surfing and environmentalism is probably linked to surfing's relation to the countercultures of the late 1960s. If this is correct, it might also to contribute to understanding of why climate protection is less on the agenda than pollution and nature conservation for ecological inclined surfers since climate change was not part of the environmental concern for the countercultures in the 1960s.

When present-day surfers are socialised in the surf culture, they learn, among other things, that as surfers, they should be concerned about the environment, *and* they should travel. Both have value and offer credibility and recognition. The action–attitude gap seems to be an inherent part of the value systems of surf cultures. In addition to regarding this gap as just a cognitive problem, we consider it as a form of *cultural dissonance*. Cultural dissonance is a concept that is used to some extent within sociology. Usually, it used to highlight disagreement *between* different cultures and philosophical traditions (Ade-Ojo and Duckworth, 2016). We use the concept in another meaning. The way we have come to use it, on the basis of the empirical material in this article, the concepts aim at understanding opposing values *within* a culture. In our instance, cultural dissonance is when surfers are given recognition for both having environmental attitudes *and* extended travelling. Both can be seen as being part of the doxa of surfing, but it is not necessarily felt as a cognitive dissonance. Rather, both travelling and consumption of surf-related goods, on one hand, and being “green,” on the other hand, are perceived as natural and to a large degree unquestioned aspects of being surfers. Even if these values can be viewed as opposing, it is still taken for granted that as a surfer, you should strive for both; you ought to be green and you should explore and be adventurous.

## Conclusion

In this study, we have aimed to explore surfers' attitudes and actions regarding environmental issues. We have found that a majority of the surfers in this study perceive themselves as environmentally conscious. Our findings also show that regarding environmental actions, surfers are mostly involved in what can be called low-cost activities that do not disrupt their identities and passions as surfers. This means that there is a gap between surfers' attitudes and actions. This gap gives rise to ambivalent feelings; many of the surfers in our study reveal that they experience what Festinger (1957) calls cognitive dissonance. To compensate for these ambivalent feelings, the surfers participate in beach clean-ups, eat less meat and state that the act of surfing and being in nature make them more prone to take environmental action.

However, our main point in this article has been to show that the attitude–action gap might be inherent to surf cultures. In surf cultures, there seems to be a cultural dissonance, opposing values that give surfers the impetus to consider themselves “green,” on one hand, and to take actions that are not environmentally sustainable, on the other hand. Hill and Abbott (2009a, p. 293) state that an organised, critical eye on the surf industry and on the destructive practises inherent in the surf culture is needed for surfing to become sustainable. We agree, and we would add that it is the doxa of surfing itself that needs to be changed. If surfers are open to such a transformation—that is an open question….

## Data Availability Statement

The raw data supporting the conclusions of this article will be made available by the authors, without undue reservation.

## Ethics Statement

The studies involving human participants were reviewed and approved by NSD (Norwegian centre for research data). The patients/participants provided their written informed consent to participate in this study.

## Author Contributions

TL and AV contributed to the conception and design of the study. AV organised and collected the data material. TL wrote the first draft of the manuscript. All authors contributed to manuscript revision, read, and approved the submitted version.

## Conflict of Interest

The authors declare that the research was conducted in the absence of any commercial or financial relationships that could be construed as a potential conflict of interest.

## Publisher's Note

All claims expressed in this article are solely those of the authors and do not necessarily represent those of their affiliated organizations, or those of the publisher, the editors and the reviewers. Any product that may be evaluated in this article, or claim that may be made by its manufacturer, is not guaranteed or endorsed by the publisher.
